# Comparably high retention and low relapse rates in different subpopulations of bipolar patients in a German non-interventional study

**DOI:** 10.1186/1471-244X-13-193

**Published:** 2013-07-17

**Authors:** Susanne Kraemer, Anette Minarzyk, Steffen Eppendorfer, Carsten Henneges, Hans-Peter Hundemer, Stefan Wilhelm, Heinz Grunze

**Affiliations:** 1 Lilly Deutschland GmbH, Medical Department, Werner-Reimers-Str. 2-4, 61352 Bad Homburg, Germany; 2 Newcastle University; Institute of Neuroscience, Newcastle, UK

**Keywords:** Bipolar disorder, Retention, Relapse, Out-patient setting, Medication regimen

## Abstract

**Background:**

Although a range of pharmacotherapeutical options are available for the treatment of bipolar disorder, patient non-adherence to prescribed treatment regimens and early treatment discontinuation remain among the primary obstacles to effective treatment. Therefore, this observational study assessed time on mood stabilizing medication and retention rates in patients with bipolar disorder (BD).

**Methods:**

In an 18-month, prospective, multicenter, non-interventional study conducted in Germany 761 outpatients (≥18 years) with BD and on maintenance therapy were documented. For analysis, patients were stratified by baseline medication: monotherapy olanzapine (OM, N = 186), lithium (LM, N = 152), anticonvulsants (N = 216), other mood stabilizing medication (OMS, N = 44); combination therapy olanzapine/lithium (N = 47), olanzapine/anticonvulsant (N = 68), other combinations (OC, N = 48). Continuation on medication was assessed as retention rates with 95% confidence intervals. Time to discontinuation and relapse-free time were calculated by Kaplan-Meier analysis. A relapse was defined as increase to CGI-BP >3, worsening of CGI-BP by ≥2 points, hospitalization or death related to BD. A Cox regression was calculated for the discontinuation of mood stabilizing therapy (reference: OM). Logistic regression models with stepwise forward selection were used to explore possible predictors of maintenance of treatment and relapse.

**Results:**

After 540 days (18 months), the overall retention rate of baseline medication was 87.7%, without notable differences between the cohorts. The overall mean time on mood stabilizing treatment was 444.7 days, with a range of 377.5 (OMS) to 481 (LM) by cohort. 74.0% of all patients were without relapse, with rates between the cohorts ranging from 58.4% (OC) to 80.2% (LM).

**Conclusions:**

Retention rates exceeded controlled trial results in all treatment cohorts, in addition to other explanations possibly reflecting that the physicians were expertly adapting treatment regimens to the individual patient’s disease characteristics and special needs.

## Background

Bipolar disorder (BD) is a common illness characterized by recurrent episodes of mania and depression, as well as mixed episodes. In the year 2000, BD ranked high in the burden of disease in disability adjusted life-years (DALYs) according to WHO [1].

In the same year in Germany a total of 343,500 days in hospital were necessary due to a primary diagnosis of bipolar disorder (ICD F31). In addition, in indirect costs 370,000 days of sick leave were documented [2]. Suicide rates are alarmingly high in bipolar patients (up to 20%) [3]. As the disease is not only of high socio-economic significance but as well of high pertinence for the patients’ personal well-being, reliable and agreeable pharmacological relapse prevention is needed [3]. Though pharmacotherapy is available for the treatment of bipolar disorder, patient non-adherence to prescribed treatment regimens and early treatment discontinuation [4,5] remain among the primary obstacles to effective treatment [6]. Keck et al [7] found 52.8% of initially hospitalized patients with mania or mixed episodes were partially or totally non-adherent to their medication at the end of a 1 year longitudinal open study (N = 106). Observations from a Danish register-based cohort study indicate significantly lower hospitalization rates for bipolar patients on lithium compared with the anticonvulsants valproate [8] or lamotrigine [9] started on relapse prevention treatment when acutely ill, while no difference was seen in patients who started it while in remission. The studies of Keck et al [7,10] were mainly focused on lithium alone. But the use of antipsychotics for maintenance therapy of bipolar disorder has been investigated as well [11-13].

Guidelines for the treatment of bipolar disorder [6,14-16] emphasize the importance of maintenance therapy with mood stabilizers. However, randomized controlled trials only partially reflect the complexity of treating patients with bipolar disorder in everyday clinical practice, which is highlighted by high relapse and discontinuation rates in this type of study [17].

To address this gap, we designed a prospective non-interventional study with the primary objective to assess time on mood stabilizing therapy and retention rates in naturalistic settings.

## Methods

### Study objectives

The primary objective of our study conducted by Lilly Germany was to assess the time on different mood stabilizing medications and retention rates in standard clinical care. Further objectives addressed relapse rates, patient adherence, and tolerability.

### Sample size estimation

At the time of protocol development, German market research reported rates of approximately 30% olanzapine use as a mood-stabilizing therapy. Based on this, it was planned to distribute 600 data collection forms to about 100 study sites. With an estimated return rate of 80%, 500 patients were planned to be documented, with approximately 150 patients treated with olanzapine and 350 patients with other medications. Tohen et al [17] found retention rates of 47% for patients treated with olanzapine and 33% for patients treated with lithium in an 18-month clinical trial for relapse prevention in bipolar disorder. Assuming a similar range of retention rates, two-sided 95% confidence intervals of the cohorts in the present study would have a range of about 8% (olanzapine, N = 150) and about 4.9% (other treatments including lithium, N = 350). Thus, the targeted sample size of 500 patients seemed to be adequate for achieving the main objective of assessing time on mood stabilizing medication and retention rates in patients with bipolar disorder over a period of 18 months.

### Study design and participants

This 18-month, prospective, multicenter, non-interventional study was conducted by hospital and office based psychiatrists throughout Germany between November 2004 and July 2007. Outpatients aged ≥18 years were enrolled if they 1) had experienced a bipolar episode within the last 6 months prior to the start of the study, which was successfully treated to remission, and 2) the patient required pharmacological maintenance treatment for mood stabilization. Remission was defined as a Clinical Global Impression-Bipolar (CGI-BP) score of ≤3 [18,19]. In line with the observational design of the study, treatment decisions were entirely at the physicians’ and the patients’ discretion. No other in- or exclusion criteria were defined; in particular having other psychiatric diagnoses besides bipolar disease was not regarded an exclusion criterion as in clinical practice patients often are treated according to their most prominent symptoms, but suffer from additional diseases, as well. No specific medication strategy was asked for by the study protocol. But it should be noted that at the time of data collection olanzapine was already established in the treatment of bipolar disorder, while other antipsychotic medications like quetiapine or aripiprazole had not yet been approved for the treatment of bipolar disorder. Written informed consent for the release of clinical data was obtained from all patients enrolled. The study was approved by the responsible ethical review board (of Medical Faculty of Ludwig-Maximilians Universität Munich) and conducted in accordance with the ethical standards of the Declaration of Helsinki.

After the initial documentation at baseline (Visit 1), data collection during the further course of routine clinical practice was scheduled in 7 visits at approximately 3, 6, 9, 12, 15, and 18 months or at early discontinuation.

At baseline, patient demographics and characteristics were collected, including the history of bipolar disease, working status, social environment, alcohol/substance consumption or substance abuse, and concomitant psychiatric disorders following ICD criteria [20]. Any pharmacological treatment for bipolar relapse prevention, i.e. treatment with mood stabilizers and other concomitant psychotropic medications was documented at every visit, including dosage, any change in treatment as well as time point and reasons for switch or discontinuation. Further parameters assessed over the course of the study were concomitant non-psychiatric medication, hospitalization due to psychiatric disorder, tolerability, suicidality, and solicited and unsolicited adverse events (AEs).

### Measures

The Clinical Global Impression scale – Bipolar (CGI-BP) is a general measure of illness severity adapted for bipolar disease with scores ranging from 1 (normal, not at all ill) to 7 (among the most extremely ill patients). Three different versions address manic, depressive and overall symptom severity (CGI-BP-manic, -depressive and -total, respectively). For evaluation, the CGI-BP results were dichotomized into patients with scores of ≤3 (patients in remission) and patients with scores >3 (no remission or relapse).

A CGI-BP increase to >3 after baseline, worsening of a minimum of 2 CGI-points, hospitalization due to psychiatric disorder, or death related to bipolar disorder were counted as relapse.

The Drug Attitude Inventory (DAI) was used to assess the patients’ attitude towards their medication (DAI-10, short version [21]). It is a 10-item self rating scale with a total score ranging between +10 and -10 (higher values indicating more positive attitude towards medication).

Additionally, drug adherence was estimated by the physician at every visit, judging how reliable the patients were in taking their medication: approx. 100%, 75%, 50%, 25%, 0% - or reliability not accessible.

### Safety and tolerability

Drug safety was evaluated for all patients who took at least one dose of bipolar maintenance medication. General tolerability was assessed with the CGI-Tolerability scale (CGI-T) [18] at every visit. The score ranges from 1 (no side effects) to 4 (side effects outweigh therapeutic effect). Results were dichotomized into ≤3 or >3, “no side effects” plus “side effects which do not affect the patient significantly” and “side effects which affect the patient significantly” plus “side effects that outweigh the therapeutic effect”.

All treatment-emergent adverse events (TEAEs) reported during the study were collected and coded according to the Medical Dictionary for Regulatory Activities (MedDRA). Serious adverse events (SAEs), discontinuations due to AEs, and related AEs were analyzed descriptively as appropriate. Additionally, a check list, assessing the presence and clinical relevance of solicited possible adverse reactions to mood stabilizers (according to these substances’ established safety profiles) was completed at every visit.

### Statistics

Statistical evaluation was largely descriptive for demographic and baseline analyses, including the time on mood stabilizing treatments. For quantitative variables means, medians, standard deviations, minimum, maximum, and quartiles, as well as the respective available sample size and the number of missing values were calculated. Categorical variables were described by absolute and relative frequencies (adjusted relative frequencies where appropriate). SAS (SAS Institute Inc., version 9.1.3) was used for statistical analyses.

The primary objective, treatment continuation, of this study, was measured as retention rate with two-sided 95% CIs of patients in the respective treatment cohort. Mean values for time on initial treatment were calculated for the different cohorts. Kaplan-Meier plots were created to describe the time to discontinuation of the baseline medication. To estimate the influence of type of medication on time on initial treatment, a Cox-regression model was calculated with time to discontinuation as dependent variable, considering the main cohorts and the following confounders: age (in years), sex (reference = female), employment (yes/no, reference = no), stable social environment (yes/no, reference = no), severity of last bipolar episode (CGI-BP ≤3 vs. >3, reference = ≤3), rapid cycling (yes/no, reference = no), history of psychiatric hospitalizations (number), psychiatric comorbidities (yes/no for each code, reference = no), alcohol/drug abuse (categorical [unknown and missing pooled], reference = no), BMI (in kg/m^2^), concomitant psychiatric medication at baseline (reference = no additional medication), number of previous bipolar episodes since onset of disease (number), number of previous manic, depressive and mixed episodes in the last 12 months (categories 0, 1, >1). Observations with missing values were excluded from the model.

Furthermore, in a post hoc analysis we calculated logistic regression models with stepwise forward selection to explore possible predictors of maintenance of treatment and the predictors of relapse. Cut-off for the inclusion of a variable into the reduced model was a p-value of ≤0.1. Variables included into the maintenance of medication model comprised sex, employment, stable social environment, somatic comorbidity, psycho-education, number of manic, depressive and mixed episodes, overall number of episodes, rapid cycling, CGI-BP total, manic and depressive, CGI-S psychotic symptoms, CGI-T, hospitalization, psychiatric comorbidity, alcohol consumption, adherence and DAI at baseline, age, age at first bipolar symptoms, first manic or mixed episode and first depressive episode. The same variables, with exception of hospitalization and CGI-T were used in the relapse model.

To further analyze subgroups of interest, patients were stratified into treatment cohorts by mood stabilizing therapy at baseline. Less frequent medications were grouped together by substance class and type of combination to allow for reasonable statistical analysis (cohort size). Treatment with antidepressants was not included in the cohort definition but is reported below as concomitant medication. Cohorts were formed as follows:

Olanzapine monotherapy: OM, N = 186

Lithium monotherapy: LM, N = 152

Anticonvulsant monotherapy: AM, N = 216

Olanzapine/lithium combination therapy: OLC, N = 47

Olanzapine/anticonvulsant combination therapy: OAC, N = 68

Other combinations of mood-stabilizers: OC, N = 48

Other mood stabilizing therapy: OMS, N = 44

At Visit 1 64.91% of all patients received concomitant psychiatric medication. OC comprises any combination containing at least one of the following: olanzapine, lithium or anticonvulsant (lamotrigine, valproic acid, carbamazepine, oxcarbazepine). OMS patients received other substances which were given aiming for mood-stabilization (like risperidone, quetiapine, clozapine, amisulpride).

## Results

### Patient disposition and early discontinuation

Patients could be included into the study at the investigator’s discretion. Overall, 761 patients were documented and evaluated. Of these, 545 (71.6%) patients reached the endpoint at 18 months. Early discontinuation was reported in 216 (28.4%) patients. Overall, the most frequent reasons for discontinuation were lost to follow-up (63 patients, 8.3%) and patient decision (30 patients, 3.9%). For 114 (15.0%) patients no reason for early discontinuation was specified. For the three largest cohorts AM, OM, and LM reasons for switching to another mood stabilizing therapy due to adverse events were reported by investigators for 13 (6.02%) AM patients, 12 (6.45%) OM patients, and 13 (5.26%) LM patients. Lack of efficacy was the reason to discontinue treatment for 25 (13.89%) AM, 20 (10.75%) OM, and 9 (5.92%) LM patients. For 30 (13.89%) AM, 29 (15.59%) OM, and 3 (1.97%) LM patients discontinuations were reported as subject decision. No switch was reported for 122 patients (56.48%) in the AM, for 99 patients (53.23%) in the OM, and 101 (66.45%) in the LM cohort. The combination therapies are not listed here as switches may be due to multi-factorial reasons. Additionally, each of these groups had less than 70 patients, being a minor fraction of the dataset.

### Baseline demographics and characteristics

The mean age of patients was 48.0 years. The age ranged between 18 and 83, with 50% (Q25 - Q75) aged between 39 and 56 years 434 (57%) patients of all patients (N = 761) were female. OLC was the only cohort with a majority of males (86 patients, 61.7%). About one third of the patients were in employment, most patients lived in a stable social environment (678 patients; 89.1%). 267 (35.1%) patients reported alcohol consumption, 31 (4.1%) alcohol abuse or addiction, and 20 (2.6%) the consumption of illicit drugs.

A history of diabetes mellitus was found in 34 (4.5%) patients, lipid metabolism disorder in 56 (7.4%), and cardiovascular disease in 77 (10.1%) patients. Overall, 220 (28.9%) patients were diagnosed with additional psychiatric disorders according to ICD-10 [20]. Of those, the most frequent diagnostic groups were: disorders of adult personality and behavior [F60–69]: (65 patients, 8.8%), neurotic, stress-related and somatoform disorders [F40-48]: (63 patients, 8.6%), mental and behavioral disorders due to psychoactive substance use [F10-19]: (42 patients, 5.7%) and schizophrenia, schizotypal and delusional disorders [F20–29]: (33 patients, 4.5%). In the different cohorts, the rate of concomitant psychiatric disorders ranged from 51.2% (n = 22) in the OMS cohort to 17.7% (n = 12) in the OAC cohort. The mean number of bipolar episodes since start of the disorder by cohort ranged from 8 to 10, except in OC (mean of 111 bipolar episodes, median 10), which comprised one ultra-rapid cycler who was reported with a number of 2000 episodes (confirmed by treating psychiatrist). In addition, there were 76 (10.0%) patients with rapid cycling. The mean numbers of manic, depressive and mixed episodes in the overall sample within the last 12 months were 1.1, 1.7 and 0.8, respectively. Thirty six point four percent of the patients had been hospitalized due to psychiatric disorders during the 12 months prior to the study, with rates ranging between 45.5% (OMS) and 30.3% (LM). On average, patients had been treated with mood stabilizers for 3.9 years (SD 4.5) before entering the study. In the treatment cohorts, this time span ranged from 2.1 years (OM) to 7.2 years (OC). One quarter in the LM group had been on at least one mood stabilizer for more than 10 years (upper quartile 10.1 years). A history of suicide attempts was reported for 193 (25.4%) patients, with rates of 37.5% (n = 18) seen in OC and of 17.0% (n = 8) in OLC. Further details of the baseline characteristics by treatment cohorts are given in Table 1.

**Table 1:** Baseline demographics and characteristics

**Variable**	**Total sample N = 761**	**OM N = 186**	**LM N = 152**	**AM N = 216**	**OLC N = 47**	**OAC N = 68**	**OMS N = 44**	**OC N = 48**
**Continuous variables**	**mean (SD)**	**mean (SD)**	**mean (SD)**	**mean (SD)**	**mean (SD)**	**mean (SD)**	**mean (SD)**	**mean (SD)**
Age [years]	48.0 (12.7)	47.8 (13.4)	50.5 (13.5)	46.4 (12.2)	48.9 (11.1)	48.0 (13.0)	44.8 (12.3)	49.7 (9.1)
BMI [kg/m^2^]	26.9 (4.7)	26.4 (4.2)	26.6 (4.2)	27.4 (5.5)	27.5 (4.1)	26.9 (4.9)	25.8 (3.7)	27.4 (4.9)
Age at first symptoms [years]	31.3 (11.5)	31.5 (11.2)	32.9 (12.7)	30.4 (11.4)	32.4 (10.1)	31.3 (10.5)	28.4 (11.6)	31.5 (11.4)
BP-episodes since start of disease [mean (SD) median]	15.5 (97.6) 6*	8.3 (16.8) 5	8.6 (9.3) 6	10.2 (11.7) 7	10.4 (10.4) 6	8.2 (7.4) 6	8.9 (6.7) 7.5	111.1 (378.7) 10*
No. of manic episodes within last 12 months	1.1 (1.2)	1.1 (1.0)	0.9 (1.2)	0.9 (1.1)	1.0 (1.2)	1.2 (1.1)	1.7 (1.5)	1.5 (1.6)
No. of depressive episodes within last 12 months	1.7 (1.3)	1.7 (1.3)	1.5 (1.3)	1.7 (1.3)	1.6 (1.4)	1.6 (1.4)	1.9 (1.3)	2.4 (1.6)
No. of mixed episodes within last 12 months	0.8 (1.3)	0.7 (1.2)	0.5 (0.9)	0.7 (1.2)	0.8 (1.4)	0.8 (1.1)	1.1 (1.6)	1.7 (2.0)
Time on mood stabilizing therapy [years]	3.9 (4.5)	2.1 (1.4)	6.7 (6.1)	2.9 (2.4)	6.1 (6.3)	2.7 (1.8)	2.5 (2.9)	7.2 (7.8)
**Binary variables**	**n (%)**	**n (%)**	**n (%)**	**n (%)**	**n (%)**	**n (%)**	**n (%)**	**n (%)**
Sex, male	327 (43.0)	86 (46.2)	73 (48.0)	75 (34.7)	29 (61.7)	31 (45.6)	16 (36.4)	17 (35.4)
Paid employment	264 (34.7)	61 (33.2)	49 (32.9)	84 (39.6)	18 (38.3)	24 (36.9)	14 (32.6)	14 (29.2)
Stable social environment	678 (89.1)	165 (89.7)	141 (94.0)	193 (91.0)	45 (95.7)	55 (84.6)	39 (90.7)	40 (83.3)
Rapid cycling	76 (10.0)	15 (8.9)	17 (11.7)	20 (9.5)	0 (0.0)	6 (9.8)	4 (9.3)	14 (33.3)
Hospitalized due to psychiatric disease (last 12 months)	277 (36.4)	71 (38.2)	46 (30.3)	75 (34.7)	20 (42.6)	25 (36.8)	20 (45.5)	20 (41.7)
Further psychiatric diseases	220 (28.9)	55 (30.4)	37 (26.2)	66 (31.1)	10 (21.7)	12 (17.7)	22 (51.2)	18 (39.1)
Alcohol consumption	267 (35.1)	53 (28.5)	67 (44.1)	71 (32.9)	18 (38.3)	20 (29.4)	18 (40.9)	20 (41.7)
Alcohol abuse or addiction	31 (4.07)	8 (1.05)	6 (0.79)	6 (0.79)	3 (0.39)	2 (0.26)	1 (0.13)	5 (0.66)
Illicit drug use	20 (2.6)	2 (1.1)	6 (3.9)	8 (3.7)	2 (4.3)	0 (0)	2 (4.5)	0 (0)
History of suicide attempts	193 (25.4)	40 (21.5)	46 (30.3)	46 (21.3)	8 (17.0)	22 (32.4)	13 (29.5)	18 (37.5)

Abbreviations: *BMI* body mass index, *BP* bipolar, *SD* standard deviation, *OM* olanzapine monotherapy, *LM* llithium monotherapy, *AM* anticonvulsant monotherapy, *OLC* olanzapine-lithium combination therapy, *OAC* olanzapine-anticonvulsant combination therapy, *OMS* other mood stabilizing therapy, *OC* other combinations, *N* number of patients.

*High mean and SD due to ultra rapid-cycling patient with 2000 episodes confirmed by investigator.

### Time on mood stabilizing treatment

After 540 days (18 months, corresponding to Visit 7) 87.7% patients of the overall sample were still on the same mood-stabilizing medication they received at baseline (CI 85.1; 90.0). The respective retention rates for the cohorts were: OM 88.1% (CI 82.1; 92.1), LM 93.6% (CI 88.0; 97.0), AM 83.8% (CI 77.8; 88.3), OLC 87.1% (CI 73.4; 94.0), OAC 86.8% (75.2; 93.1), OC 84.2% (CI 69.7; 92.2), OMS 85.9% (CI 71.3; 93.4). Concomitant antidepressant medication was prescribed to 41.65% of all patients at Visit1 and to 32.77% on Visit 7 (V7). At V1 18.13% received selective serotonin reuptake inhibitors (SSRIs), 12.48% tricyclic antidepressants (TCAs), and 8.41% serotonin/ noradrenaline reuptake inhibitors (SNRIs).

The median (Q1/Q3) time on mood stabilizing treatment, calculated from baseline, ranged between 484.0 (OMS) and 538.0 (OLC) days (Table 2). Overall, the median time to a change of treatment was 525.0 days (374.0/554.0). Patients on lithium (534.0 days, 485.5/553.5) stayed on their medication longer than those from the cohorts OM (519.5 days, 335.0/548.0) and OMS (484.0 days, 230.0/531.0).

**Table 2:** Time on mood stabilizing treatment since baseline and retention rates during the study

**Cohort**	**N**	**Mean [days]**	**95% CI [days]**	**SD [days]**	**1st Quartile [days]**	**Median [days]**	**3rd Quartile [days]**	**Retention-Rate (%)**	**95% CI (%)**
OM	186	433.5	408.4 - 458.5	173.0	335	520	548	88.1	82.1 - 92.1
LM	152	481.3	458.7 - 504.0	141.2	486	534	554	93.6	88.0 - 96.6
AM	216	440.4	416.8 - 464.0	175.8	363	527	558	83.8	77.8 - 88.3
OLC	47	475.1	428.4 - 521.8	159.1	454	538	570	87.1	73.4 - 94.0
OAC	68	417.5	371.1 - 463.9	191.8	262	514	552	86.8	75.2 - 93.2
OC	48	462.2	413.9 - 510.5	166.3	403	529	568	84.2	69.7; 92.2
OMS	44	377.5	318.8 - 436.2	193.1	230	484	531	85.9	71.3; 93.4
Total sample	761	444.7	432.5 - 456.9	171.2	374	525	554	87.7	85.1 - 90.0

Abbreviations: *OM* Olanzapine monotherapy, *LM* Lithium monotherapy, *AM* Anticonvulsive monotherapy, *OLC* Olanzapine/lithium combination therapy, *OAC* Olanzapine/anticonvulsive combination therapy, *OC* Other combinations of mood-stabilizers, *OMS* Other mood stabilizing therapy, *SD* standard deviation, *CI* confidence interval.

Start date was start of documentation. The end date was date of either the first discontinuation or switch of mood stabilizing medication, death, or the last documented visit.

After 540 days (18 months), 87.7% of patients of the overall sample were still on the same mood stabilizing medication they had been taking at baseline (95% CI 85.1; 90.0).

### Primary analysis

**Figure 1:**
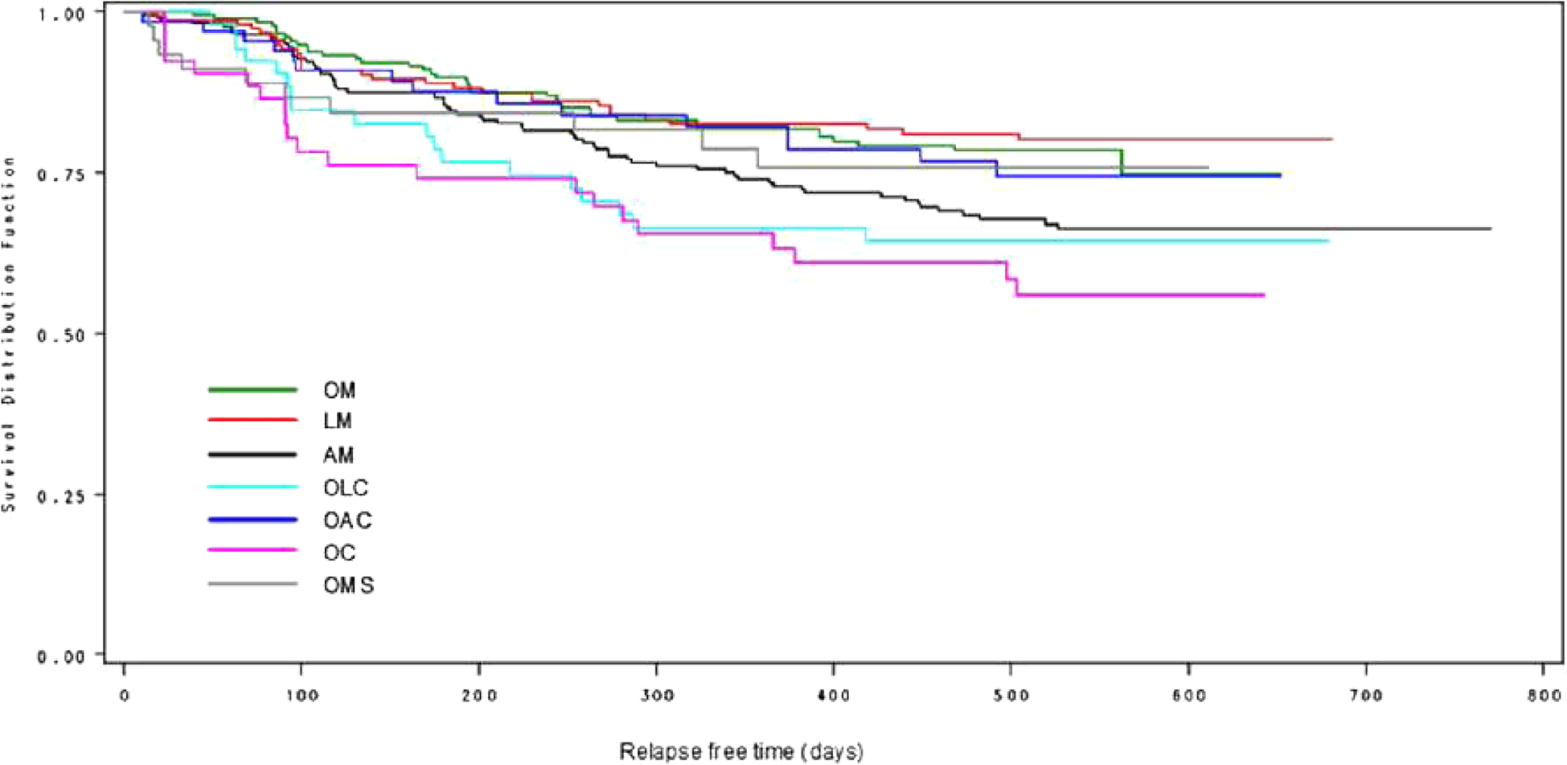
**Kaplan-Meier plot for total relapse-free-time by treatment cohort with change of treatment considered censored.** All patients (N = 761). Definition of relapse: CGI-BP worsening of a minimum of 2 points, hospitalization due to psychiatric disease, and death related to bipolar disease were counted as an event (i.e. relapse). Patients who dropped out or died from other causes and patients who changed their mood stabilizing therapy were considered censored. Abbreviations: OM = Olanzapine monotherapy, LM = Lithium monotherapy, AM = Anticonvulsive monotherapy, OLC = Olanzapine/lithium combination therapy, OAC = Olanzapine/anticonvulsive combination therapy, OC = Other combinations of mood-stabilizers, OMS = Other mood stabilizing therapy.

Figure 1 shows the Kaplan-Maier curves for time to relapse for the cohorts. A CGI-BP worsening of a minimum of 2 points, a CGI-BP increase to >3 after baseline, hospitalization due to psychiatric disorders, and death related to bipolar disorder were regarded as an event (i.e. relapse). Patients who dropped out or died from other causes were considered censored. Furthermore, patients were considered censored if they changed their mood stabilizing therapy. At Visit 7 (18 months), 74.0% of the patients in the overall sample were without relapse. The percentages for the cohorts were: OM 78.5% (95% CI 71.5; 84.0), LM 80.2% (95% CI 72.7; 85.8), AM 68.9% (95% CI 62.0; 74.9), OLC 64.2% (95% CI 49.3; 75.8), OAC 74.4% (95% CI 61.0; 83.8), OC 58.4% (95% CI 43.1; 71.0), OMS 78.7% (95% CI 62.8; 88.4).

The proportion of patients without hospitalization due to psychiatric disorders while in the study ranged from 69.5% (OC) to 90.7% (OM) with 95% CI for the observed differences ranging between 53.8 - 80.8 and 85.0 - 94.3, respectively.

### Duration on mood stabilizing treatment

**Figure 2:**
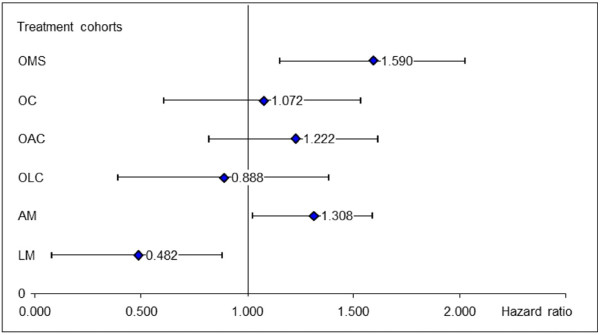
**Cox regression: Hazard ratios of treatment cohorts for the discontinuation of primary mood stabilizing therapy with olanzapine monotherapy (OM) as reference.** Abbreviations: OM = Olanzapine monotherapy, LM = Lithium monotherapy, AM = Anticonvulsive monotherapy, OLC = Olanzapine/lithium combination therapy, OAC = Olanzapine/anticonvulsive combination therapy, OC = Other combinations of mood-stabilizers, OMS = Other mood stabilizing therapy.

**Figure 3:**
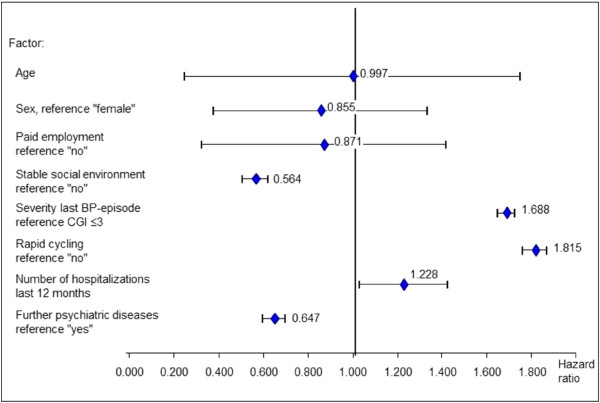
**Risk for discontinuation of primary mood stabilizing therapy.** Association with solicited factors (Cox). Abbreviations: OM = Olanzapine monotherapy, LM = Lithium monotherapy, AM = Anticonvulsive monotherapy, OLC = Olanzapine/lithium combination therapy, OAC = Olanzapine/anticonvulsive combination therapy, OC = Other combinations of mood-stabilizers, OMS = Other mood stabilizing therapy.

The Cox- proportional hazard regression (Figure 2, with OM as reference) shows that patients treated with OMS and AM were more likely to discontinue their medication than in OM. Patients treated with LM had about a 2-fold chance to continue their treatment compared to patients treated with OM. Factors associated with a greater risk of discontinuing the primary mood stabilizing therapy were living in a non-stable social environment, last bipolar episode more severe, comorbid psychiatric diseases, and rapid cycling (Figure 3).

At baseline, the mean DAI score was 5.8 (95% CI 5.5 to 6.1) for the overall sample. The score increased to 6.8 (95% CI 6.5 to 7.1) after 18 months of observation. By cohorts the mean DAI score ranged between 5.1 (OMS) and 6.9 (OAC) at baseline and between 6.2 (OMS) and 8.2 (OAC) at 18 months. At baseline the DAI (95% CI) was lower for OM (5.65; 5.07, 6.22) compared to OLC (6.87, 5.94, 7.80) and OAC (6.89; 6.02, 7.75). The mean DAI score improved for all cohorts during the course of the study. At 18 months the DAI was higher for OAC (8.20; 7.52, 8.87) compared to OM (6.72; 6.09, 7.34), LM (6.41; 5.65, 7.17) and AM (6.60; 6.09, 7.11).

Factors significantly associated with treatment maintenance were alcohol consumption (advantage for consumption), baseline adherence (advantage for good baseline adherence), number of manic episodes (advantage for less episodes) and baseline CGI-BP (advantage low CGI ≤3). (Details of the model are given in Table 3).

**Table 3:** Factors significantly associated with maintenance of treatment

**Factor**	**Odds ratio**	**95% CI**	**p-value**
Baseline adherence (no vs. yes)	0.013	0.001 – 0.146	0.0004
Manic episodes (no vs. >1)	12.953	2.773 – 60.504	0.0011
Manic episodes (1 vs. >1)	8.209	2.131 – 31.620	0.0022
CGI BP total (≤3 vs. >3)	10.971	2.018 – 59.651	0.0056
Alcohol consumption (any vs. no)	4.563	1.287 – 16.181	0.0188

Abbreviations: *CGI BP* Clinical Global Impression Score Bipolar, *CI* confidence interval.

### Remission, stability and relapses

The CGI-BP score was dichotomized into patients with a CGI-BP-total of ≤3 (criterion of remission) and patients with a CGI-BP >3 (criterion of relapse after baseline). At baseline, 642 (84.5%) patients fulfilled the criterion of remission, which implies that 118 (15.5%) patients actually had not met one of the inclusion criteria. However, a sensitivity check, excluding these patients, revealed that this hardly affected the overall results.

At Visit 7, 459 (85.0%) patients had a CGI-BP total of ≤3. The respective rates in the cohorts were similar at both visits. The logistic regression model yielded a higher number of depressive episodes, higher baseline CGI-BP total score and higher CGI-BP depressive scores at baseline (see Table 4) as predictors for relapse.

**Table 4:** Factors significantly associated with relapse

**Factor**	**Odds ratio**	**95% CI**	**p-value**
CGI BP total (≤3 vs. >3)	0.118	0.046 – 0.302	<0.0001
CGI BP last depressive episode (≤3 vs. >3)	3.642	1.506 – 8.804	0.0041
No. of depressive episodes (1 vs. >1)	3.401	1.407– 8.220	0.0066
Duration of depressive episodes (per year)	0.967	0.935 – 1.000	0.0473

Abbreviations: *CGI BP* Clinical Global Impression Score Bipolar, *CI* confidence interval.

### Safety and tolerability

#### ***CGI-tolerability***

Poor tolerability (CGI-tolerability score ≥3) was reported by 55 (7.6%) patients at baseline and by 28 (5.3%) at Visit 7. In the cohorts the rates ranged between 6.2% (OM, OAC) and 13.3% (OLC) at baseline and between 3.1% (OC) and 8.3% (OMS) at Visit 7.

#### ***Treatment-emergent adverse events***

Overall, TEAEs were reported by 113 (14.9%) patients, 78 of these (10.3%) had AEs classified as related to medication. Eight patients were reported with SAEs: one patient from the LM cohort experienced a lithium overdose which was the only SAE considered to be related to the mood stabilizing therapy. The same patient was also reported with syncope. Other SAEs were: 1 appendectomy, 1 breast cancer, 1 retinal detachment and 4 deaths. 2 women from the OC cohort, 47 and 64 years old, died during the observational period. No further information could be obtained for the circumstances of death in these patients. One 67-year old man from the AM cohort died of myocardial infarction, and one 54 year old woman from the OLC cohort committed suicide. This latter patient was in acute mania and was reported as having insufficient adherence. None of these cases was considered related to the mood stabilizing therapy by the treating study physician, though, due to lack of information, this cannot be ruled out completely for the two fatalities in the OC cohort. An overview on TEAEs in the cohorts is given in Table 5.

**Table 5:** Treatment-emergent adverse events (TEAEs), overview of all patients (N = 761)

**Patients with:**	**Total sample N = 761**		**OM N = 186**		**LM N = 152**		**AM N = 216**		**OLC N = 47**		**OAC N = 68**		**OC N = 48**		**OMS N = 44**	
	**N**	**%**	**N**	**%**	**N**	**%**	**N**	**%**	**N**	**%**	**N**	**%**	**N**	**%**	**N**	**%**
Any TEAE	113	14.9	21	11.3	27	17.8	31	14.4	9	19.2	8	11.8	7	14.6	10	22.7
Related TEAE	78	10.3	14	7.5	19	12.5	23	10.7	6	12.8	6	8.8	3	6.3	7	15.9
Any SAE	8	1.1	0	0.0	3	2.0	1	0.5	1	2.1	0	0.0	2	4.2	1	2.3
Related SAE	1	0.1	0	0.0	1	0.7	0	0.0	0	0.0	0	0.0	0	0.0	0	0.0
TEAE leading to discontinuation	7	0.9	0	0.0	2	1.3	1	0.5	1	2.1	0	0.0	2	4.2	1	2.3
Lethal outcome	4	0.5	0	0.0	0	0.0	1	0.5	1	2.1	0	0.0	2	4.2	0	0.0

Abbreviations: *N* number of patients, *OM* Olanzapine monotherapy, *LM* Lithium monotherapy, *AM* Anticonvulsive monotherapy, *OLC* Olanzapine/lithium combination therapy, *OAC* Olanzapine/anticonvulsive combination therapy, *OC* Other combinations of mood-stabilizers, *OMS* Other mood stabilizing therapy, *TEAE* treatment emergent adverse events, *SAE* serious adverse events.

Weight gain as TEAE was reported in 42 (5.5%) patients (OM 12; 6.5%, LM 7; 4.6%, AM 9; 4.2%, OLC 3; 6.4%, OAC 3; 4.4%, OC 2; 4.2%, OMS 6; 13.6%), tremor in 13 (1.7%) patients (LM 5; 3.3%, AM 3; 1.4%, OLC 3; 6.4%, OC 1; 2.1%, OMS 1; 2.3%). Vomiting occurred in 3 (1.4%) patients of the AM cohort. All other types of TEAEs were reported with rates <1% of the overall sample, and occurring in <3 patients per cohort.

#### ***Solicited adverse reactions***

Six hundred and sixty-two (87.0%) patients reported at least one adverse event from the solicited list at least once in the course of the study. The proportion of patients reporting solicited adverse reaction in the cohorts ranged from 95.7 (OLC) to 79.6 (OMS). The adverse reaction reported most frequently throughout all cohorts was weight increase, with rates ranging from 89.4% (OLC) to 59.1% (OMS). Details are given in Table 6. Weight gain and sedation were more prevalent in cohorts with olanzapine mono- or combination therapy, while cohorts receiving lithium mono- or combination therapy had higher rates of tremor, thirst, and gastrointestinal complaints.

**Table 6:** Descriptive analysis of patients with solicited adverse events, all patients (N = 761)

	**All Patients N = 761**		**OM N = 186**		**LM N = 152**		**AM N = 216**		**OLC N = 47**		**OAC N = 68**		**OC N =48**		**OMS N = 44**	
**Solicited side effects**	**n**	**%**	**n**	**%**	**n**	**%**	**n**	**%**	**n**	**%**	**n**	**%**	**n**	**%**	**n**	**%**
Any	662	87.0	157	84.4	135	88.8	185	85.7	45	95.7	61	89.7	44	91.7	35	79.6
Weight increase	530	69.7	144	77.4	99	65.1	140	64.8	42	89.4	49	72.1	30	62.5	26	59.1
Sedation	369	48.5	100	53.8	56	36.8	101	46.8	27	57.5	37	54.4	23	47.9	25	56.8
Cognitive dysfunctions	321	42.2	74	39.8	68	44.7	78	36.1	25	53.2	36	52.9	26	54.2	14	31.8
Sexual dysfunctions	294	38.6	78	41.9	48	31.6	79	36.6	30	63.8	33	48.5	15	31.3	11	25.0
Gastroint. disorders	221	29.0	40	21.5	58	38.2	59	27.3	16	34.0	15	22.1	19	39.6	14	31.8
Dizziness	215	28.3	46	24.7	44	29.0	64	29.6	13	27.7	19	27.9	15	31.3	14	31.8
Tremor	195	25.6	25	13.4	62	40.8	35	16.2	22	46.8	23	33.8	22	45.8	6	13.6
Thirst	169	22.2	30	16.1	58	38.2	36	16.7	18	38.3	12	17.7	10	20.8	5	11.4
Insomnia	165	21.7	41	22.0	33	21.7	46	21.3	12	25.5	12	17.7	14	29.2	7	15.9
Polyuria/nycturia	109	14.3	21	11.3	49	32.2	16	7.4	12	25.5	5	7.4	4	8.3	2	4.6
Menstrual disorders	65	8.5	16	8.6	11	7.2	23	10.7	2	4.3	6	8.8	4	8.3	3	6.8
Thyroid disorders	58	7.6	5	2.7	31	20.4	7	3.2	7	14.9	2	2.9	6	12.5	0	0.0
Akathisia	54	7.1	12	6.5	7	4.6	18	8.3	1	2.1	9	13.2	3	6.3	4	9.1
Parkinson syndrome	21	2.8	3	1.6	5	3.3	6	2.8	1	2.1	3	4.4	2	4.2	1	2.3
Hyperprolactinaemia	18	2.4	3	1.6	5	3.3	3	1.4	3	6.4	3	4.4	0	0.0	1	2.3
Tardive dyskinesia	16	2.1	1	0.5	4	2.6	7	3.2	1	2.1	1	1.5	2	4.2	0	0.0
Dystonia	16	2.1	2	1.1	2	1.3	5	2.3	1	2.1	0	0.0	3	6.3	3	6.8
QTc-Prolongation	8	1.1	0	0.0	3	2.0	3	1.4	0	0.0	1	1.5	1	2.1	0	0.0

Abbreviations: *N* number of patients, *OM* Olanzapine monotherapy, *LM* Lithium monotherapy, *AM* Anticonvulsive monotherapy, *OLC* Olanzapine/lithium combination therapy, *OAC* Olanzapine/anticonvulsive combination therapy, *OC* Other combinations of mood-stabilizers, *OMS* Other mood stabilizing therapy.

## Discussion

Though the majority of the patients were of the same age group (i.e. middle-aged), considerable diversity in the characteristics of their disease was observed (i.e., duration, severity, number, type of episodes). In contrast to randomized trials, the composition of the therapy cohorts in this study is diverse: the baseline data suggest that the regimens chosen are the result of differential considerations of the treating psychiatrists according to their patients’ history and needs [22].

The observed discontinuation rate of 28.4% in our study was low compared to rates observed in controlled clinical trials [13,17]. Furthermore, we found comparably high retention rates in all treatment cohorts, even though the patients in these cohorts differed perceptibly in clinical preconditions. In general, patients on lithium tended to retain their medication longer, and discontinued less often than patients in any of the other cohorts. As lithium is one of the oldest and most established medications for the treatment of bipolar disease, a high percentage of patients in this group has been taking this medication for a long period of time (some >10 years). As patients would hardly retain a medication this long if they were not comfortable with it, the implication is that in the lithium group we observe a pre-selection of rather satisfied and therefore compliant patients. Kessing et al [23] in 2007 published data from a Danish medical register study with the finding for lithium that the mean time to discontinuation was 181 days. The discrepancy in time on medication to our observation might again be due to the patient population on a long standing lithium medication, who had passed the time point of early discontinuation found in the same publication to be at 45.2 days for 25% of patients.

In contrast, the OC and OMS cohorts comprised more difficult-to-treat patients: patients with mixed episodes, rapid cycling, and low adherence. Especially, OMS patients tended to suffer more often from additional psychiatric illnesses, experience more hospitalizations, less clinical improvement, reach remission status less frequently, switch to new medications earlier, and have a less positive attitude towards their medication.

The various treatment cohorts were largely comparable with regard to time on mood stabilizing medication. Though the longest time on mood stabilizing therapy was seen in LM, the difference was only notably large compared to OM and OMS. No further notable differences were observed between the cohorts. In addition, there was no relevant difference at all in the retention rates. The hazard for discontinuation was also comparable in most cohorts. Only in LM the risk was lower than in OM. In AM and OMS it was higher than in OM. This observation, as well as other factors found associated with increased risk of discontinuation (non-stable social environment, comorbid psychiatric diseases, and rapid cycling) matches well with the notion that OMS comprised patients who were rather difficult to treat. A similar but less pronounced tendency could be observed for the OC cohort. It should be noted that in this cohort with combinations of less frequently used mood stabilizing agents, the highest proportion of patients with rapid cycling was found.

The drug attitude measured as DAI was positive and high from the start in all cohorts, indicating that the majority of these outpatients had a positive attitude regarding their medication at the start of the study. It improved only slightly over the course of the study. This might result from several factors: 1) the selection criteria of stabilization, 2) the natural treatment setting where physicians are familiar with their patients through a long-lasting therapeutic relationship, and 3) increased adherence through the patients’ awareness of taking part in a study. Quite in line with this, good baseline adherence was found to be one predictor of adherence throughout the study, while a higher number of manic episodes and a high baseline CGI were predictors of non-adherence: OMS had the highest number of manic episodes, the highest percentage of patients with a baseline CGI-BP >3, and the lowest DAI.

Interestingly, alcohol consumption was positively associated with adherence. The variable in the model was “any consumption”, and 35.1% of the patients reported consumption, whereas only 4.1% stated alcohol abuse or addiction. One could speculate, that this observation possibly could reflect the occasional social drink, which might be an indicator of social integration presumably associated with general well-being and stability.

The majority of patients were without relapse at Visit 7 (day 540), rates between the cohorts were largely similar. The only notable difference was seen in OC compared to OM and LM which corresponds with the predictors found in the logistic regression model. OC had indeed the highest number of depressive episodes and also the highest number of patients with a CGI-BP >3.

As a CGI-BP of ≤3 was an inclusion criterion, there was little room for improvement, and thus the CGI-BP scores decreased only slightly over time in all cohorts. Correspondingly, the rates of patients with a CGI-BP ≤3 were around 80% to 90% in all cohorts at both visits, with only minor variations in both directions. The aim of the maintenance treatment, i.e. stabilization, could thus be regarded as achieved.

Due to the observational design of the study and the determination of the mood stabilizing medication that was chosen for the individual patient according to clinical reasoning, a comparison between the different cohorts of medications and combinations is not intended and not possible. We should stress the point that patient enrollment to the study was at the discretion of the treating physician. Therefore, results have to be interpreted in the context that medication and patients were selected on the basis of their individual preferences and history. In summary, the different treatments we observed achieved similar effects regarding maintenance treatment of bipolar disease. This is surprising regarding the diversity of the patients’ disease and social characteristics at the start of documentation, hinting again at the capability of physicians and their patients to optimize individual treatment using the spectrum of medications available. Thus, this trial design mimics clinical treatment reality better than a randomized study taking into account physicians’ skills and patient diversity. Besides the classic treatments with lithium and anticonvulsants, olanzapine was found to be a relevant mood stabilizing treatment option for a considerable number of patients in this German study sample. Other atypical antipsychotics were also used in clinical practice, but as these had not yet been approved for the treatment of bipolar disease at the start of the study, they were applied in few patients only (OMS-cohort).

Only small numbers of patients were reported with TEAEs or adverse events as assessed by the CGI-tolerability.

On the other hand, 87.0% of the overall sample reported at least one adverse event at least once over the course of the study on the predefined solicited checklist of side effects specific for the various mood stabilizing medications. Obviously, these effects were observed, but not judged by the patients to be relevant enough to be reported spontaneously as AEs. Solicited adverse events also were no reason for physicians or patients to stop or switch medication.

Within the cohorts, OLC patients reported the highest overall number and variety of solicited adverse events, closely followed by the OC groups (which also included patients receiving lithium plus one or several other substances for mood stabilization). Compared to LM it appears that these patients experienced adverse events of lithium as well as typical ones for the other mood stabilizers. In all cohorts, the most common AE was weight gain. This underlines the potential risk of weight gain in the maintenance therapy of bipolar disorder, the need to monitor and if necessary to treat bipolar patients for metabolic-related adverse events.

## Conclusion

The data from this observational study in Germany show that a wide range of medication is used to provide individual mood stabilizing therapies to single patients.

The finding that physicians and their patients achieved comparably satisfactory results, despite diversity in disease and individual patients shows that medications are used by physicians with great knowledge and with focus on the individual patient and the needs of clinical practice.

Beside the classical mood stabilizers like lithium or anticonvulsants there is an increasing use of atypical antipsychotic medications like olanzapine in the maintenance therapy of bipolar disorders. Combination seems to be chosen mainly for difficult to treat patients with a high burden of disease.

### Limitations

Several limitations should be considered in this study performed by Lilly Germany: The treatment regimens were not randomized, but chosen by the treating psychiatrists according to the patient’s individual need, which created selection bias in the way that we see patients with more severe symptoms accumulate in some groups while other cohorts comprise predominately the more stable ones. In addition, the protocol did not advise the physicians to enroll patients consecutively, which could possibly have led to some additional selection bias.

Patients were stratified into treatment cohorts post hoc. Considering that there had been no restriction regarding the medication applied for mood stabilization, a large variety of substances was employed, but only few in a frequency which allowed the formation of monotherapy cohorts with patient numbers large enough for reasonable evaluation. The remainder therefore had to be grouped by substance type and type of combination. Even then there remained considerable differences in sample sizes per treatment cohort; hence these differences should be considered with caution.

At the time when this study was started, olanzapine was the only antipsychotic approved for maintenance treatment of bipolar disease in Germany. It was therefore the only substance of this class which was used frequently enough to form a substantial cohort. However we saw other antipsychotics applied as mood stabilizers in clinical practice, which were not yet approved in Germany for this indication, but for which some evidence had been generated at the time that they might be effective. As a consequence, these were employed infrequently, seemingly as a last resort, in cases especially difficult to treat (e.g. rapid cyclers, patients with mixed episodes).

## Competing interests

The authors S. Kraemer, S. Eppendorfer, C. Henneges, HP Hundemer, S. Wilhelm are empoyees of Lilly Deutschland GmbH. A. Minarzyk had been employee of Lilly Germany at the time of study execution and drafting of manuscript. H. Grunze received grants / research support, consulting fees and honoraria within the last three years from Astra Zeneca, BMS, Desitin, Eli Lilly, Gedeon-Richter, Hoffmann-LaRoche, Janssen-Cilag, Lundbeck, Merck, Otsuka, Sanofi-Aventis, Servier, Sepracor, and UBC.

## Authors’ contributions

HPH and HG authored the study protocol with input from SE. SW was responsible for study execution; SK for study execution and the statistical analysis plan; CH for statistics; AM drafted the manuscript. All authors were continuously involved in writing the manuscript, and read and approved the final manuscript.

## Pre-publication history

The pre-publication history for this paper can be accessed here:

http://www.biomedcentral.com/1471-244X/13/193/prepub
